# Highly Efficient Photo-Fenton Ag/Fe_2_O_3_/BiOI Z-Scheme Heterojunction for the Promoted Degradation of Tetracycline

**DOI:** 10.3390/nano13131991

**Published:** 2023-07-01

**Authors:** Jingjing Zheng, Guoxia Liu, Zhengbo Jiao

**Affiliations:** 1 Department of Chemical Engineering and Safety, Binzhou University, Binzhou 256603, China; zjj65050@163.com (J.Z.); liuguoxialgx@163.com (G.L.); 2 Institute of Materials for Energy and Environment, Qingdao University, Qingdao 266071, China

**Keywords:** photo-Fenton, Z-scheme-heterojunction, Ag, BiOI, Fe_2_O_3_

## Abstract

Novel Ag/Fe_2_O_3_/BiOI Z-scheme heterostructures are first fabricated through a facile hydrothermal method. The composition and properties of as-synthesized Ag/Fe_2_O_3_/BiOI nanocomposites are characterized by powder X-ray diffraction, scanning electron microscopy, high-resolution transmission electron microscopy, UV-Vis diffuse reflectance spectra, etc. The Ag/Fe_2_O_3_/BiOI systems exhibit remarkable degradation performance for tetracycline (TC). In particular, the composite (Ag/Fe_2_O_3_/BiOI-2) shows the highest efficiency when the contents of Ag and α-Fe_2_O_3_ are 2 wt% and 15%, respectively. The effects of operating parameters, including the solution pH, H_2_O_2_ concentration, TC concentration, and catalyst concentration, on the degradation efficiency are investigated. The photo-Fenton mechanism is studied, and the results indicated that •O^2−^ is the main active specie for TC degradation. The enhanced performance of Ag/Fe_2_O_3_/BiOI heterostructures may be ascribed to the synergic effect between photocatalysis and the Fenton reaction. The formation of Ag/Fe_2_O_3_/BiOI heterojunction is beneficial to the transfer and separation of charge carriers. The photo-generated electrons accelerate the Fe^2+^/Fe^3+^ cycle and create the reductive reaction of H_2_O_2_. This research reveals that the Ag/Fe_2_O_3_/BiOI composite possesses great potential in wastewater treatment.

## 1. Introduction

Wastewater is a major threat to human health, and some organic pollutants are highly toxic even in low concentrations [1,2]. Tetracycline (TC) is a kind of antibiotic and is important to medical treatment and animal husbandry. However, its long-term residual in water will result in adverse effects on water resources and human health [3,4]. As a kind of advanced oxidation process (AOPs), the heterogeneous Fenton process is widely used in pollutants treatment due to its low toxicity, environmental compatibility, and low cost [5,6,7]. However, its reaction efficiency is low due to the poor cyclic ability of Fe^3+^/Fe^2+^ on the catalyst surface. Improving the conversion rate of Fe^3+^ to Fe^2+^ is essential to pollutant degradation performance [8,9]. Fenton-like systems under visible light irradiation have been found to exhibit high efficiency and relatively high mineralization [10]. With the introduction of light, an accelerated Fe^2+^/Fe^3+^ cycle is achieved in the traditional Fenton process, as the accumulated Fe^3+^ is reduced effectively by photo-generated electrons from the photocatalysts under light irradiation [11]. Therefore, it is highly desirable to design novel photo-Fenton catalysts with superior performance. Combining semiconductor materials with various iron-based materials is attracting more and more attention in improving the conversion rate of Fe^3+^ to Fe^2+^ in the visible-light-driven photo-Fenton system, such as FeOOH/g-C_3_N_4_, Cu-FeOOH/g-C_3_N_4,_ and Fe_2_O_3_/BiOBr [12,13,14]. Bismuth oxyiodide (BiOI) is a layered structure of (Bi_2_O_2_)^2+^ layers interleaved with double slabs of I atoms with a band gap of 1.77–1.92 eV and thus can absorb visible light [15]. However, the rapid recombination of photo-generated carriers makes the BiOI exhibit poor photocatalytic activity [16]. The preparation of the heterogeneous photocatalyst has been proven to be an efficient way to address this issue [17,18]. Fe_2_O_3_, a kind of magnetic material, is generally thermally stable, low-cost, and readily available. It also has high corrosion resistance. It has been widely explored in the process of environment protection, such as heavy metal ion adsorbents, photocatalysts, and so on [19,20,21]. The band gap of Fe_2_O_3_ is about 2.1 eV, similar to that of the BiOI. So, they can easily form a heterojunction with a matching band [22,23]. In the past several years, many ternary heterojunction photocatalysts containing nano-Ag particles were structured, including BiOI/Ag/AgI, Ag/BiOI/ZnFe_2_O_4_, Ag/BiOI/rGO, Ag/Ag_2_O/BiVO_4,_ and so on [24,25,26,27]. The high photocatalytic activities of these ternary heterojunctions are mainly ascribed to both the heterojunction structure and the effect of nano-Ag particles [28]. The surface plasmon resonance effect (SPR) of silver (Ag) nanoparticles (NPs) enhances the visible-light absorption capacity of heterojunction. Meanwhile, the Ag NPs could effectively separate and transfer the photo-generated carrier. Precious metal loading is one of the effective ways to increase the photo-efficiency of photocatalytic materials. However, until now, the fabrication and the photo-Fenton activities of the ternary composites with Fe_2_O_3,_ BiOI, and nano-Ag NPs have not been reported.

In this study, we fabricate a novel Ag-doped Fe_2_O_3_/BiOI ternary heterojunction through a hydrothermal-co-precipitation method. A series of Ag/Fe_2_O_3_/BiOI photo-Fenton materials with different content of BiOI have been prepared for comparison. Tetracycline is adopted as the simulated pollutant to evaluate the photo-Fenton performance of Ag/Fe_2_O_3_/BiOI in the presence of H_2_O_2_ under visible light (≥420 nm). The as-synthesized Ag/Fe_2_O_3_/BiOI ternary heterojunctions exhibit higher photo-Fenton performance than the Fe_2_O_3_ and BiOI. Furthermore, a possible mechanism for the enhanced photo-Fenton performance of the Ag/Fe_2_O_3_/BiOI composite is proposed.

## 2. Materials and Methods

### 2.1. Materials

All chemical materials were used devoid of additional purification. Ferric nitrate hydrate (Fe(NO_3_)_3_·9H_2_O, ≥99.0%, Tianjin Tianli chemical reagent Co., Ltd., Tianjin, China), aquae hydrogenii dioxidi (H_2_O_2_, 30%, Tianjin Tianli chemical reagent Co., Ltd., Tianjin, China), ethylene glycol ((HOCH_2_)_2_, EG, Tianjin Beifang chemical reagent Co., Ltd., Tianjin, China), bismuth nitrate pentahydrate (Bi(NO_3_)_3_·5H_2_O, ≥99.0%, Sinopharm Chemical Reagent Co., Ltd., Shanghai, China), silver nitrate (AgNO_3,_ ≥99.0%, Tianjin Hengxing Chemical reagent Co., Ltd., Tianjin, China), potassium iodide (KI, ≥99.0%, Sinopharm Chemical Reagent Co., Ltd., Shanghai, China), urea (CO(NH_2_)_2_, AR, Tianjin Zhiyuan chemical reagent Co., Ltd., Tianjin, China), and tetracycline (TC, ≥99.0%, Sinopharm Chemical Reagent Co., Ltd., Shanghai, China). The water utilized in all experiments was deionized water, and the whole experiment process was carried out at room temperature.

### 2.2. Preparation of Fe_2_O_3_ Nanoparticles, Ag/Fe_2_O_3_ Composite and Ag/Fe_2_O_3_/BiOI Heterojunction

To prepare Fe_2_O_3_ nanoparticles, firstly, 2 g Fe(NO_3_)_3_·9H_2_O was dissolved into 100 mL deionized water and stirred for 15 min. Subsequently, 2 g urea was gradually added to the Fe(NO_3_)_3_ solution under vigorous stirring, and then the mixture was magnetically stirred for 15 min. The mixture was put into a Teflon-lined stainless steel autoclave of 100 mL and heated at 180 °C for 12 h. After the reaction was completed, the product was rinsed with deionized water and ethanol three times, respectively. Finally, it was dried at 60 °C overnight.

A total of 2 mmol of Fe_2_O_3_ was added into 20 mL ultrapure water under fierce stirring to obtain the Fe_2_O_3_ suspension. The aqueous solution of AgNO_3_ (5 mL) was dripped into the Fe_2_O_3_ suspension. The suspension was stirred for 30 min and then illuminated under a 300 W Xe lamp for 1 h to reduce the silver element. The obtained sample was collected and washed with deionized water as well as ethanol. Finally, the sample was dried at 60 °C overnight. In this work, several Ag/Fe_2_O_3_ composites with the different molar ratios of Ag were prepared.

To prepare Ag/Fe_2_O_3_/BiOI, firstly, Bi(NO_3_)_3_·5H_2_O (0.388 g, 0.8 mmol) was dispersed in 100 mL ethylene glycol with constant stirring to obtain the well-dispersed solution. An amount of Ag/Fe_2_O_3_ and KI (0.0528 g, 0.8 mmol) was dispersed into 30 mL of ethylene glycol under fierce stirring to obtain the homogeneous Ag/Fe_2_O_3_/KI suspension, respectively. Then, the Bi(NO_3_)_3_ solution was dripped into the Ag/Fe_2_O_3_/KI suspension. Subsequently, this mixture was put into a Teflon-lined stainless steel autoclave of 100 mL and heated at 90 °C for 12 h and cooled to room temperature. The obtained products were filtered, washed with water and ethanol several times, and dried at 100 °C under vacuum for 12 h. The final products were denoted as Ag/Fe_2_O_3_/BiOI-1, Ag/Fe_2_O_3_/BiOI-2, Ag/Fe_2_O_3_/BiOI-3, and Ag/Fe_2_O_3_/BiOI-4, the mass percent of Ag was 2%, and the mass percent of Fe_2_O_3_ was 10%, 15%, 20%, and 25% respectively. In this work, several Ag/Fe_2_O_3_/BiOI composites with different mass percent of Ag were also prepared, which were denoted as Ag/Fe_2_O_3_/BiOI (1%), Ag/Fe_2_O_3_/BiOI (2%), Ag/Fe_2_O_3_/BiOI (4%), and Ag/Fe_2_O_3_/BiOI (8%), the mass percent of Fe_2_O_3_ was 15%, and the mass percent of Ag was 1%, 2%, 4%, and 8% respectively. For comparison, BiOI was also synthesized with the same procedure without the addition of Ag/Fe_2_O_3_.

### 2.3. Characterization

Powder X-ray diffraction was documented on a Bruke AXS D8 Advance powder diffractometer with CuKα radiation (λ = 0.15406 nm). Field emission scanning electron microscope (FE-SEM) images were recorded to gain the morphology of samples on a Hitachi S-4800. High-resolution transmission electron microscopy (HRTEM) images were recorded to gain morphology and microstructures of the synthesized samples on the JOEL JEM-2100 microscope. UV-Vis diffuse reflectance spectra (DRS) were recorded on the UV-Vis-NIR spectrophotometer (Varian Cary 5000). X-ray photoelectron spectroscopy (XPS) was done on an Escalab 250Xi X-ray photoelectron spectrometer with an Al Ka excitation source to analyze the chemical composition and chemical state of the synthesized samples. Magnetic measurements were recorded at room temperature by a physical property measurement system (PPMS) and vibrating sample magnetometer (VSM) (Quantum Design, Beijing, China).

### 2.4. Photo-Fenton Activity Measurement

The photo-Fenton performance activity of the synthesized samples was evaluated by degrading 100 mL of TC solution (10 mg/L) in the presence of a 100 mg catalyst. The initial pH (pH = 6.8) was adjusted by 0.1 mol/L HNO_3_ or NaOH. The suspensions were kept in the dark for 30 min to reach the adsorption-desorption equilibrium, and then the suspensions were added with 10 mM H_2_O_2_ and exposed to a 300 W Xe lamp with a 420 nm cut-off filter. About 3 mL aliquots were collected and centrifuged immediately every 4 min. The absorbance of the solution was then measured at 357 nm on a Shimadzu UV-2550UV-Vis spectrometer to analyze the degradation of the TC solution. The formula for calculating the photocatalytic degradation efficiency was as follows:

Photodegradation efficiency (%) = (C_0_ − C_t_)/C_0_ × 100%

where t represented time, C represented the concentration of TC, C_0_ was the initial concentration of TC, and C_t_ was the concentration of TC at time t.

The H_2_O_2_ concentration was measured by a traditional cerium sulfate Ce(SO_4_)_2_ titration method [29].

## 3. Results and Discussion

### 3.1. Structural Characterization of Prepared Samples

**Figure 1:**
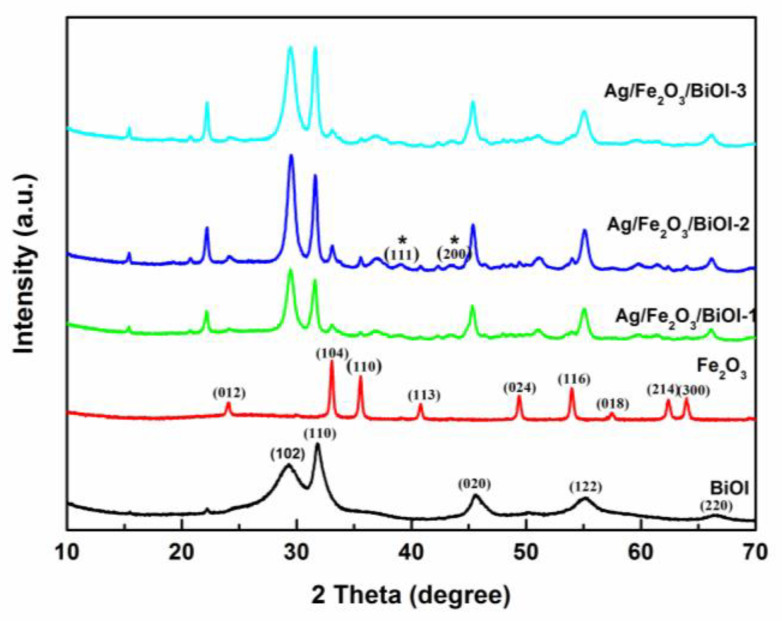
XRD patterns recorded for α-Fe_2_O_3,_ BiOI, and Ag/Fe_2_O_3_/BiOI composites. (* Reflection corresponding to the Ag phase).

The XRD phase analysis of α-Fe_2_O_3,_ BiOI, and Ag/Fe_2_O_3_/BiOI composites are presented in Figure 1. It can be seen that the characteristic diffraction peaks of α-Fe_2_O_3_ and BiOI are in good agreement with the rhombohedra α-Fe_2_O_3_ phase (JCPDS No. 33-0664) and standard tetragonal phase of BiOI (JCPDS No. 10-445), respectively [30]. No impurities are detected, and the diffraction peaks of α-Fe_2_O_3_ and BiOI are apparent in the XRD patterns of the Ag/Fe_2_O_3_/BiOI composites. The peak at 2θ = 38.1° and 44.3° in the patterns of Ag/Fe_2_O_3_/BiOI-2 (marked by stars) are found to belong to the (111) and (200) plane of the face-centered-cubic phase of silver (JCPDS 04-0783) [31]. However, the low amount of Ag resulted in weak diffraction peaks of Ag. The diffraction peaks of Ag/Fe_2_O_3_/BiOI composites suggest the coexistence of Fe_2_O_3,_ BiOI, and Ag.

### 3.2. Morphological Analysis

**Figure 2:**
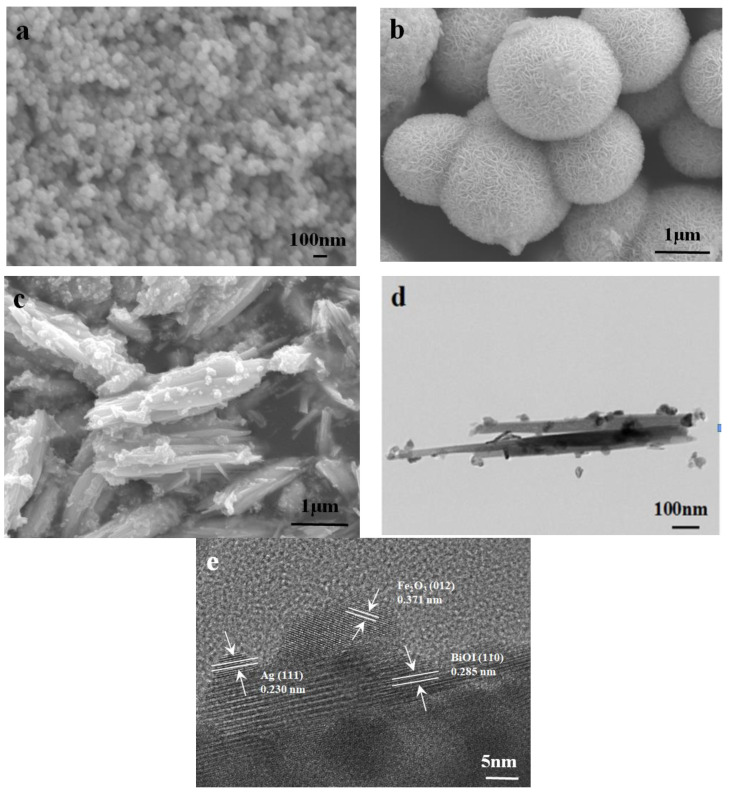
SEM images of (**a**) pristine α-Fe_2_O_3,_ (**b**) BiOI, (**c**) Ag/Fe_2_O_3_/BiOI-2, (**d**) TEM, and (**e**) HRTEM of Ag/Fe_2_O_3_/BiOI-2.

The morphologies and microstructures of pristine α-Fe_2_O_3_, BiOI, and Ag/Fe_2_O_3_/BiOI-2 are observed by SEM, TEM, and HRTEM, and the results are shown in Figure 2. As shown in Figure 2a, the α-Fe_2_O_3_ displays sphere particles of approximately 40–60 nm in diameter. Figure 2b shows that the BiOI is a sphere particle composed of irregular, smooth sheets with a diameter of about 1 μm. With regards to Ag/Fe_2_O_3_/BiOI-2 (Figure 2c), α-Fe_2_O_3_ spheres and Ag particles are deposited on the surface of the BiOI sheets. TEM images of Ag/Fe_2_O_3_/BiOI-2 (Figure 2d) are consistent with SEM images, which indicate that α-Fe_2_O_3_, Ag, and BiOI have been combined. From the HRTEM image in Figure 2e, the discontinuous lattices could be clearly observed. The lattice spacing of 0.371 nm is ascribed to the (012) plane of α-Fe_2_O_3_ [30]_._ The lattice fringe at 0.285 nm corresponds to the (110) plane of BiOI [32]. The lattice spacing of 0.230 nm is ascribed to the Ag (111) plane [33]. The above results confirm that α-Fe_2_O_3_, BiOI, and Ag have been successfully prepared and coexist in the ternary Ag/Fe_2_O_3_/BiOI composite.

### 3.3. XPS Analysis

**Figure 3:**
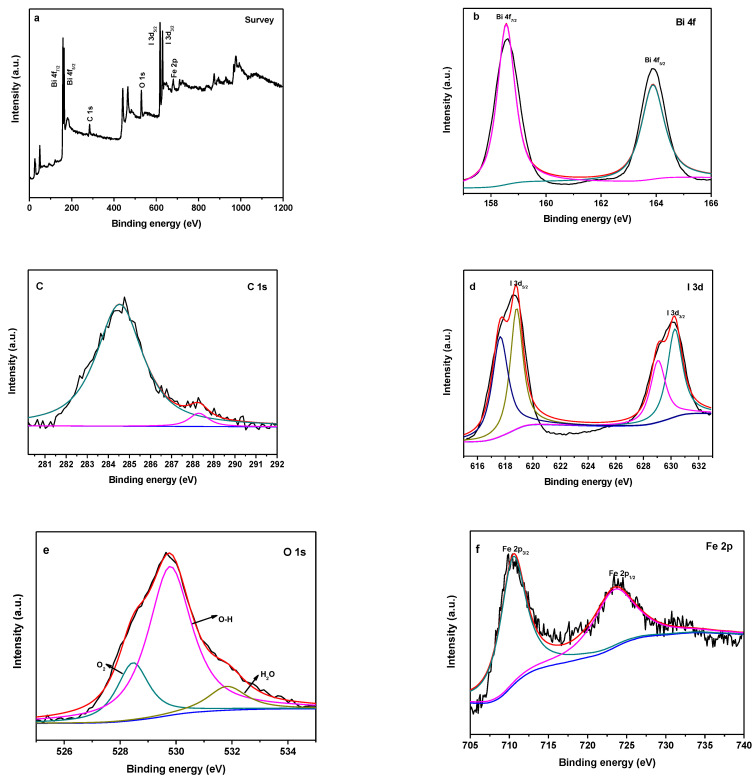

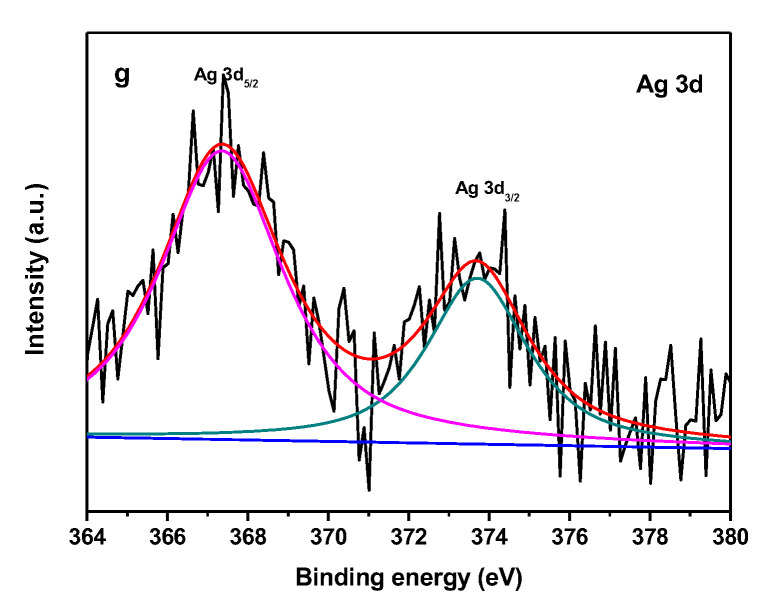
High-resolution XPS spectra of Ag/Fe_2_O_3_/BiOI-2 (**a**) survey, (**b**) Bi 4f, (**c**) C 1s, (**d**) I 3d, (**e**) O 1s, (**f**) Fe 2p, (**g**) Ag 3d.

The elemental composition and chemical states of the Ag/Fe_2_O_3_/BiOI composites are characterized by XPS (as shown in Figure 3). As presented in Figure 3a, the XPS survey spectrum indicates that the Ag/Fe_2_O_3_/BiOI composites are composed of Bi, O, I, Fe, and Ag. The atomic concentration for Ag, Fe, O, Bi, and I elements of Ag/Fe_2_O_3_/BiOI composites are shown in Table 1; as the loading ratio of Fe_2_O_3_ increased, its atomic content also increased. The high-resolution XPS spectra of Ag/Fe_2_O_3_/BiOI-2 are shown in Figure 3b–g. Figure 3b shows a high-resolution 4f spectrum of Bi in the Ag/Fe_2_O_3_/BiOI-2. The binding energies (BEs) of Bi 4f_7/2_ and Bi 4f_5/2_ are 158.4 eV and 164.1 eV, respectively, which are typical of values reported for BiOI [34]. In Figure 3c, the C 1s peak centered at 283.4 eV can be ascribed to carbon contamination [30]. In Figure 3d, the BEs of I 3d_5/2_ and I 3d_3/2_ are 617.4, 619.3, and 631.0; 629 eV, respectively, which are consistent with the I^-^ of BiOI [35]. Figure 3e shows the O 1s XPS spectrum and there are three peaks: 528.5 eV, 529.8 eV, and 531.8 eV. The peaks at 528.5 and 529.8 eV are assigned to the lattice oxygen of BiOI and Fe_2_O_3_, respectively [36,37]. The peak at 531.8 eV belongs to adsorbed water molecules [38]. Figure 3f shows the Fe 2p XPS spectrum of Ag/Fe_2_O_3_/BiOI-2. The peaks at 724.5 and 711.8 eV are assigned to the Fe 2p_1/2_ and Fe 2p_3/2_ states, respectively, which indicate the formation of α-Fe_2_O_3_ in the Ag/Fe_2_O_3_/BiOI-2 [39]. The peaks center at 367.5 and 377.6 eV in Figure 3g corresponded to Ag 3d_5/2_ and Ag 3d_3/2_, respectively [24].

### 3.4. Ultraviolet-Visible Reflectance Spectra

**Figure 4:**
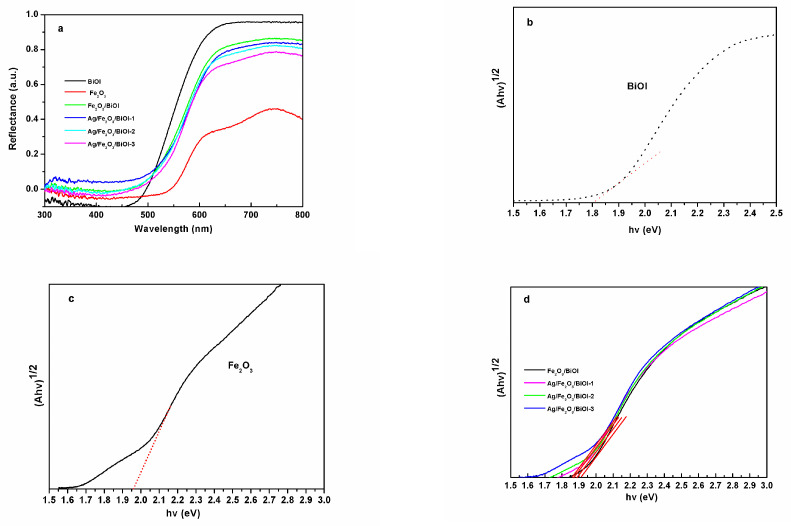
(**a**) UV-Vis DRS spectra of as-prepared samples; (**b**) plots of the (αhν)^1/2^ vs. photon energy (hν) for BiOI (the red line is tangent line) ; (**c**) α-Fe_2_O_3_ (the red line is tangent line) and (**d**) Ag/Fe_2_O_3_/BiOI composites.

The optical absorption property of photocatalysts is important to photocatalytic performance. Figure 4a is the UV-Vis diffuse reflectance spectra (DRS). It is found that the pristine α-Fe_2_O_3_ exhibits a main absorption edge at about 750 nm. The absorption edge of BiOI is about 600 nm. The Fe_2_O_3_/BiOI composite shows a blended absorption feature of pure BiOI and α-Fe_2_O_3_ and is similar to the pure BiOI. Furthermore, after the introduction of Ag nanoparticles, the Ag/Fe_2_O_3_/BiOI ternary composites display higher absorption intensity over the range from 500 to 700 nm compared with Fe_2_O_3_/BiOI.

The forbidden bandwidth is evaluated by the Kubelka-Munk equation, as shown in Figure 4b–d. The band gap of BiOI and α-Fe_2_O_3_ are 1.79 and 1.95 eV, respectively, which are close to the previous research [40,41]. The band gap of Ag/Fe_2_O_3_/BiOI-1, Ag/Fe_2_O_3_/BiOI-2, and Ag/Fe_2_O_3_/BiOI-3 are estimated as 1.86, 1.87, and 1.88 eV, respectively. The narrowing bandgaps of hybrid materials are consistent with the enhanced optical response.

### 3.5. VSM Analysis

**Figure 5:**
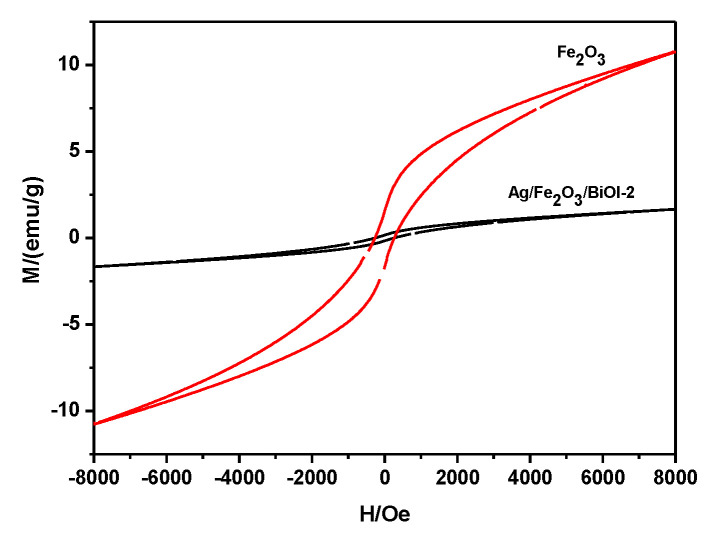
Hysteresis loops recorded at room temperature of α-Fe_2_O_3_ (the red line) and Ag/Fe_2_O_3_/BiOI-2. (the black line).

Magnetic property is measured using a vibrating sample magnetometer (VSM) at room temperature to investigate the magnetic response of Ag/Fe_2_O_3_/BiOI in the external magnetic field. The magnetic hysteresis loops of the as-prepared samples are shown in Figure 5. It can be seen that the α-Fe_2_O_3_ and Ag/Fe_2_O_3_/BiOI have superparamagnetism. The Ms values of α-Fe_2_O_3_ and Ag/Fe_2_O_3_/BiOI-2 are approximately 11.8 and 3.7 emu/g, respectively. The results demonstrate that the as-prepared Ag/Fe_2_O_3_/BiOI exhibits magnetic performance and can solve the retrieval problem of the catalyst in the suspension phase system.

### 3.6. Evaluation of the Photo-Fenton Performance

**Figure 6:**
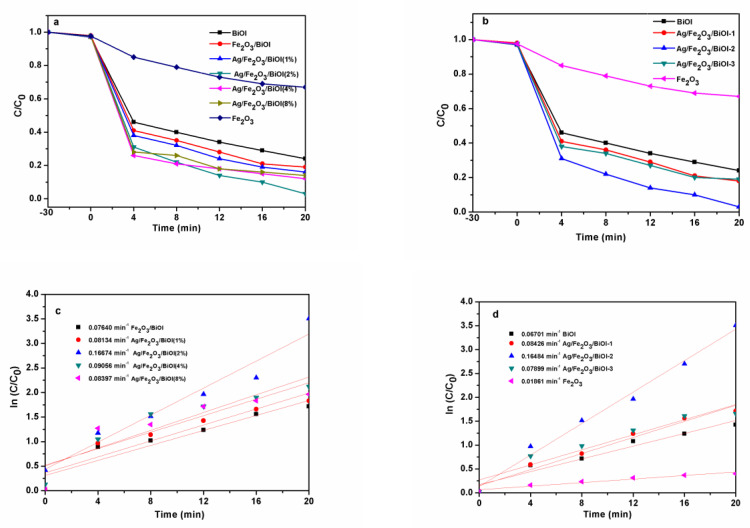
(**a**) Photo-Fenton degradation of TC on different samples (different Ag content); (**b**) photo-Fenton degradation of TC on different samples (different α-Fe_2_O_3_ content); (**c**,**d**) corresponding apparent rate constants of TC in photo-Fenton reaction. (H_2_O_2_: 10 mM; Ph: 6.5; catalyst dosage: 1 g/L; TC: 10 mg/L).

In this study, the photo-Fenton performance of the as-prepared samples is evaluated through TC degradation under visible light (Figure 6). As illustrated in Figure 6a, the TC self-degradation is almost unobserved in the absence of the catalyst within 20 min, indicating that TC is quite stable in the photo-Fenton system. As shown in Figure 6a,b, all catalysts show relatively weak adsorption capability. Without irradiation and oxidant, the TC removal rates for all catalysts are about 2%. The photo-Fenton degradation rates of TC for pristine BiOI and α-Fe_2_O_3_ are 76% and 32% in 20 min, respectively. When the Ag content is 2 wt%, the TC degradation rate of Ag/Fe_2_O_3_/BiOI (2%) is optimum. As illustrated in Figure 6b, when the α-Fe_2_O_3_ content is changed, the photo-Fenton performance of Ag/Fe_2_O_3_/BiOI first increases with increasing content of α-Fe_2_O_3_ and then reduces when the content of α-Fe_2_O_3_ reaches 15% (Ag/Fe_2_O_3_/BiOI-2). The degradation rate of Ag/Fe_2_O_3_/BiOI-2 reaches 96% after 20 min irradiation. Moreover, it is higher than BiOI, α-Fe_2_O_3,_ and other composites. This is because the light absorption of BiOI decreases due to the excess coverage of α-Fe_2_O_3_. Therefore, only when the appropriate content (15 wt%) of α-Fe_2_O_3_ and Ag (2 wt%) are coupled with BiOI, the visible-light-assisted photo-Fenton degradation of Ag/Fe_2_O_3_/BiOI-2 nanocomposites reach *a* maximum. In addition, to further explore the reaction kinetics of TC, the degradation kinetics curves are also shown in Figure 6c,d. It can be seen that the experimental data of the catalysts in the degradation process of TC match well with the first-order kinetics model. The highest decolorization rate constant value of Ag/Fe_2_O_3_/BiOI-2 is approximately eleven and three times higher than those of α-Fe_2_O_3_ and BiOI, respectively. The results confirm that an appropriate combining amount of α-Fe_2_O_3_ and Ag could result in the enhancement of the photo-Fenton activity.

**Figure 7:**
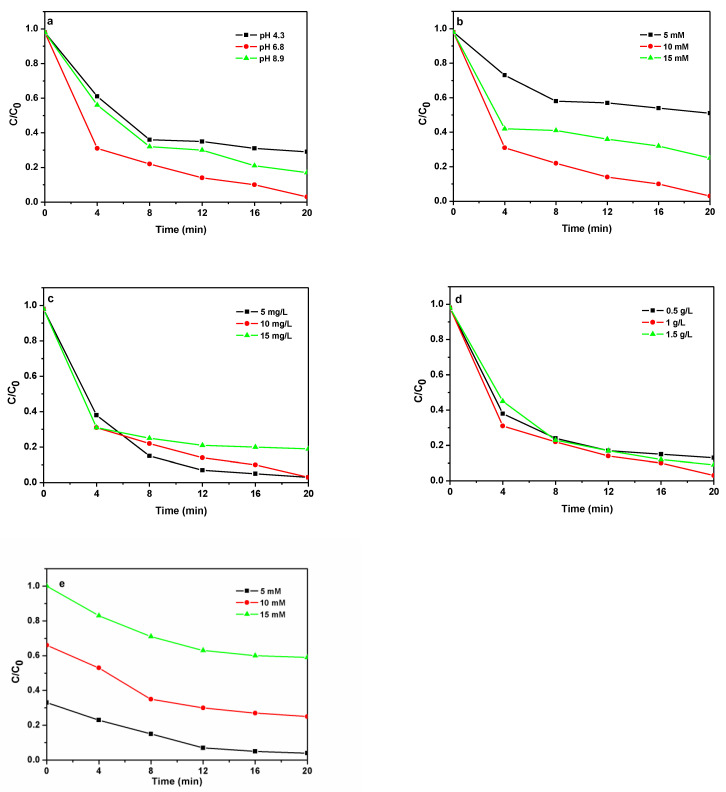
Effect of (**a**) solution pH (**b**) H_2_O_2_ concentration (**c**) TC concentration (**d**) catalyst concentration on the photo-Fenton degradation performance of Ag/Fe_2_O_3_/BiOI-2 under visible-light irradiation. (**e**) The rate of H_2_O_2_ decomposition in the process of photo-Fenton degradation. (H_2_O_2_: 10 mM; Ph: 6.5; catalyst dosage: 1 g/L; TC: 10 mg/L).

Generally, solution pH, H_2_O_2_ concentration, reagent concentration, and catalyst dosage play a great role in the application process. So, the effects of these conditions on the degradation of TC by Ag/Fe_2_O_3_/BiOI-2 are shown in Figure 7. As shown in Figure 7a, TC degradation efficiency increases with increasing pH, firstly from 4.3 to neutral conditions (6.8), and then decreases when the pH is further increased to 8.9. This indicates that the heterogeneous Fenton reaction contributes more than the homogeneous Fenton reaction in this system [30]. It can be seen that the degradation activity of Ag/Fe_2_O_3_/BiOI-2 gradually increases with H_2_O_2_ concentration from 5 to 10 mM (Figure 7b). However, the decomposition rate would decrease again if the H_2_O_2_ concentration is 15 mM, which may be owing to the adverse consumption of excess H_2_O_2_ by the free radical [42]. Figure 7c shows the influence of TC concentration on the photo-Fenton degradation activity of Ag/Fe_2_O_3_/BiOI-2. When the concentrations of TC are 5, 10 mg/L, and 15 mg/L, the TC is nearly completely degraded by Ag/Fe_2_O_3_/BiOI-2 in 20 min. But the TC concentration is increased to 20 mg/L, and 90% of TC is degraded. Figure 7d shows the degradation performances for various initial catalyst concentrations. When the Ag/Fe_2_O_3_/BiOI-2 dosage is 1 g/L, the degradation rate of TC is optimal. This is due to more catalyst dosage providing more active sites but excess catalyst blocking the penetration of light hence restricting TC degradation. In order to explain the effect of H_2_O_2_ on the decomposition of TC, the rate of H_2_O_2_ decomposition as a function of its concentration is tested. As shown in Figure 7e, the rate of H_2_O_2_ decomposition and the rate of TC decomposition are not linearly related, mainly due to the adverse consumption of excess H_2_O_2_ by the scavenging free radical effect [42].

**Figure 8:**
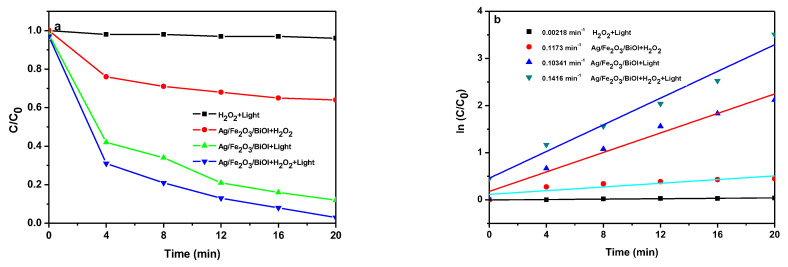
(**a**) The effect of reaction protocols on TC degradation and corresponding (**b**) apparent rate constants of TC on Ag/Fe_2_O_3_/BiOI-2. (H_2_O_2_: 10 mM; Ph: 6.5; catalyst dosage: 1 g/L; TC: 10 mg/L).

The control experiments are conducted to study the specific roles of the Fenton and photocatalytic reaction in the photo-Fenton system. As shown in Figure 8a, in the case of pure H_2_O_2_ under visible light irradiation, the degradation of TC is almost unobserved within 15 min. In this study, the Ag/Fe_2_O_3_/BiOI-2 presents a removal efficiency of about 85.5% by the visible-light irradiation of 20 min, and the Ag/Fe_2_O_3_/BiOI-2 and H_2_O_2_ present a removal efficiency of about 33.5% within 20 min. Furthermore, Ag/Fe_2_O_3_/BiOI-2 and H_2_O_2_ under visible-light irradiation exhibit the highest photo-Fenton catalytic efficiency (95%). The reaction rate constants of these reactions in the TC degradation are shown in Figure 8b. The rate constant of the Fenton reaction, photocatalytic reaction, and photo-Fenton reaction are 0.1416 min^−1^, 0.1034 min^−1,^ and 0.0173 min^−1^, respectively.

### 3.7. Catalyst Stability

**Figure 9:**
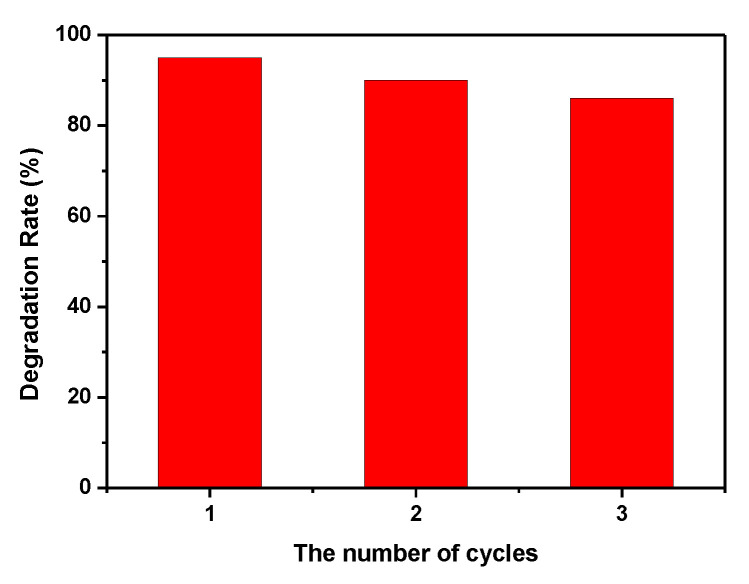
Recycling test of Ag/Fe_2_O_3_/BiOI-2 in photo-Fenton degradation of TC.

The lifetime is very important for a catalyst in practical application. Therefore, it is evaluated by the circulation experiments in this study. As shown in Figure 9, the photo-Fenton catalytic activity of Ag/Fe_2_O_3_/BiOI-2 can be maintained above 85% after three recycles under identical experimental conditions. This result illustrates that the Ag/Fe_2_O_3_/BiOI has good prospects in sewage treatment because of its high performance and good long-term stability.

### 3.8. Photo-Fenton Mechanism

**Figure 10:**
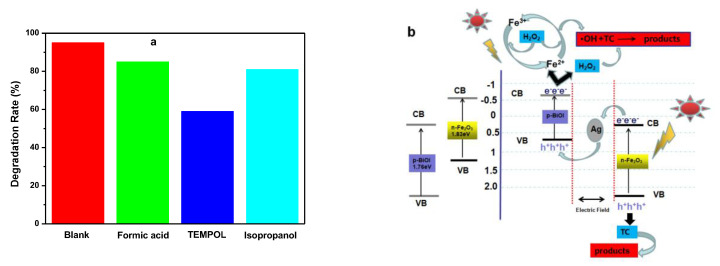
(**a**) The histograms of different scavengers on photo-Fenton reaction effect about Ag/Fe_2_O_3_/BiOI-2, (**b**) Possible mechanism of the photo-generated carrier transfer pathways in Ag/Fe_2_O_3_/BiOI under visible-light irradiation.

Figure 10a is a histogram of the effect of different scavengers on the photo-Fenton process. The degradation rates of TC are drastically reduced after adding TEMPOL (•O_2_^−^ scavenger). When the isopropanol (•OH scavenger) and formic acid (h^+^ scavenger) are added to the solution, the degradation rates of TC are slightly reduced. Compared with those without any scavenger, the degradation rate decreases from 95% to 85%, 59%, and 81%, respectively, which indicates that •O^2−^ is mainly responsible for this photo-Fenton reaction.

In order to explain the enhanced photo-Fenton performance of Ag/Fe_2_O_3_/BiOI, the band edge positions of the conduction band (CB) and valence band (VB) potentials of α-Fe_2_O_3_ and BiOI are confirmed. As a semiconductor, the CB and VB can be calculated on the basis of the empirical equation: E_CB_ = X − E_e_ − 0.5E_g_ (1); E_VB_ = E_CB_ + E_g_ (2). E_e_ is the energy of free electrons on the hydrogen scale (Ee = 4.5 eV), and X is the absolute electronegativity of the semiconductor. The X values for BiOI and α-Fe_2_O_3_ are 5.94 and 4.78, respectively [22]. Eg is the band gap energy of the semiconductor. The band gap energies of BiOI and α-Fe_2_O_3_ are 1.79 eV and 1.95 eV, respectively, according to Figure 4b,c. From the calculation, the E_CB_ of BiOI and α-Fe_2_O_3_ is 0.55 eV and −0.695 eV versus NHE, and the E_VB_ of BiOI and α-Fe_2_O_3_ are estimated to be 2.31 eV and 1.135 eV versus NHE, respectively. BiOI is a p-type semiconductor, and its Fermi level is located close to the VB. The α-Fe_2_O_3_ is an n-type semiconductor, and its Fermi level is located close to the CB [22].

Based on the above results, a photo-Fenton mechanism is proposed for the Ag/Fe_2_O_3_/BiOI. As shown in Figure 10b, both BiOI and α-Fe_2_O_3_ can be excited under visible-light irradiation. When BiOI and α-Fe_2_O_3_ are combined to form a p-n junction, the electrons generated from the CB of Fe_2_O_3_ will transfer to the CB of BiOI and accumulate near the junction in p-BiOI. Meanwhile, the holes generated from the VB of BiOI will diffuse to the VB of Fe_2_O_3_ and enrich Fe_2_O_3_ near the junction. With the equilibration of Fermi levels of BiOI and Fe_2_O_3_, an internal electric field is built to stop the transfer of electrons. The energy band of BiOI is raised up, and that of Fe_2_O_3_ is shifted downwards along with the Fermi level [22]. Therefore, the CB bottom of BiOI is higher than that of Fe_2_O_3_. It was therefore proposed that the electrons generated in the CB of Fe_2_O_3_ transfer to the VB of BiOI and combine with photoexcited holes to accumulate the electrons in the CB of BiOI and holes in the VB of Fe_2_O_3_. Ag nanoparticles are deposited on the interface of Fe_2_O_3_/BiOI heterojunction and function as an electroconductive bridge to form the visible-light-driven Ag/Fe_2_O_3_/BiOI Z-scheme system, which plays an important role in electron transfer. So, when the heterojunction Ag/Fe_2_O_3_/BiOI is exposed to visible light (λ > 420 nm) and Ag NPs are paired between Fe_2_O_3_ and BiOI, the electrons generated from CB of Fe_2_O_3_ would transfer to the VB of BiOI. The CB potential of Fe_2_O_3_ was more negative than that of Ag due to its high Schottky barriers at the interface of the semiconductor-metal; thus, the electrons generated from the CB of Fe_2_O_3_ would transfer to metallic Ag and combine with the holes of BiOI. So the electrons of Fe_2_O_3_ and holes of BiOI would combine through Ag nanoparticles. As a result, the electron shift effectively blocks the recombination of electron-hole pairs and prolongs the lifetime of free electrons and holes. Moreover, the SPR effect of Ag nanoparticles can enhance visible-light absorption capacity and rapid separation and transportation of photo-generated electron holes. So when the Ag/Fe_2_O_3_/BiOI composites are exposed to visible light, a lot of photo-generated electrons and holes are generated. The photo-generated electrons accelerate the Fe^2+^/Fe^3+^ cycle and create the reductive reaction of H_2_O_2_. The holes can directly oxidize organic molecules and create the oxidative reaction of H_2_O_2_. The possible reaction formula is shown below:

Fe^3+^ + H_2_O_2_ → Fe^2+^ + •O_2_^−^ + 2H^+^

Fe^2+^ + H_2_O_2_ → Fe^3+^ + •OH + OH^−^

Fe^3+^ + e^−^ → Fe^2+^

h^+^ + H_2_O_2_ → •O_2_^−^ + 2H^+^

H_2_O_2_ + e^−^ → •OH + OH^−^

So the increased degradation effect of Ag/Fe_2_O_3_/BiOI is owing to the synergic effect between photocatalysis and Fenton reaction [43].

## 4. Conclusions

To sum up, the novel Ag/Fe_2_O_3_/BiOI Z-scheme p-n heterojunctions can be easily synthesized through a facile hydrothermal method. The Ag/Fe_2_O_3_/BiOI heterostructures exhibit good photo-Fenton activity for the degradation of TC via the activation of H_2_O_2_.

Furthermore, the photo-Fenton mechanism is further studied, and the results show that •O^2−^ is the main active species. The study results indicate that the Ag/Fe_2_O_3_/BiOI heterojunction facilitates the transfer and separation of photo-generated electron-hole pairs and accelerates the Fe^2+^/Fe^3+^ cycle. The combination of efficient electron separation and the solid-liquid interfacial heterogeneous Fenton process is the reason for the good photo-Fenton degradation performance of Ag/Fe_2_O_3_/BiOI. Thus it will be a potential way to reform Z-scheme p-n heterojunctions in order to improve photo-Fenton catalysis technology for practical large-scale applications.

## Figures and Tables

**Table 1:** Atomic concentration for Ag, Fe, O, Bi, and I elements of Ag/Fe_2_O_3_/BiOI composites.

Samples	Ag (at.%)	Fe (at.%)	O (at.%)	Bi (at.%)	I (at.%)
Ag/Fe_2_O_3_/BiOI-1	0.19	2.36	14.91	10.72	71.82
Ag/Fe_2_O_3_/BiOI-2	0.20	3.47	21.89	9.67	64.77
Ag/Fe_2_O_3_/BiOI-3	0.20	4.78	30.18	8.42	56.42

## Data Availability

Not applicable.
